# Influence of pore structure on humidity parameters of cement-polymer mortars contaminated with filamentous fungi

**DOI:** 10.1371/journal.pone.0231347

**Published:** 2020-04-09

**Authors:** Elżbieta Stanaszek-Tomal

**Affiliations:** Chair of Building Materials Engineering, Faculty of Civil Engineering, PK Cracow University of Technology, Cracow, Poland; Mirpur University of Science and Technology, PAKISTAN

## Abstract

Mineral building materials are porous materials. The volume of pores connected is the active volume of pores or the effective volume. The volume of all pores is the total volume of the pores. The properties of the individual pores are different. Their dimensions and shape can influence the properties of materials. Materials are modified with different admixtures to improve their properties. However, additives or admixtures can cause corrosion. Although building materials do not provide food for microorganisms, they are very often inhabited by them. As a result of their presence and the action of metabolic products, biodeterioration occurs. One of the products of metabolism is water. In this paper we investigated how the modified structure of biodeterioration caused by mould fungi affects the moisture content of cement-polymer mortar with the admixture of polysiloxane latex.

## 1. Introduction

Both technical and construction materials have a large number of empty spaces in their internal structure. Their relative size is small compared to the size of the material itself. These spaces are pores. They vary in size and shape. The term 'pores' means the class of voids connected to the outer surface. With their help, liquids penetrate into the material. However, it is common for pores that are connected to the external surface to be described as open pores. Voids that do not have this connection are referred to as closed pores. Those that do not have such a connection, however, are closed pores. Interconnected pores form a pore space in the material, usually filled with fluid, air, water and so on. The remaining fixed part is called a skeleton. The total volume of pores is the sum of the volume of open and closed pores. In the open-pore system, a distinction can be made between the connected pores, which allow the flow of fluids (transport) and the connected blind pores. The flow of liquids and gases is only possible in open pores. In such pores, fluid flow may be accompanied by heat transfer, filtration, diffusion, sorption and chemical reactions. The volume of the pores connected is the active volume of the pores or the effective volume. The volume of all pores is called the total volume of the pores [1].

There are various pore divisions in the literature. However, the most commonly used is adsorption. According to the pore size classification, introduced by the International Union of Pure and Applied Chemistry (IUPAC), three pore classes are distinguished: micropores with a width of <2 nm, mesopores with a width of 2–50 nm and macropores with a width of> 50 nm. However, the boundaries between the individual pore classes are arbitrary. They were adopted on the basis of adsorption criteria [2].

The properties of the individual pores are different. Thus, micropores and mesoporosis determine the size of the inner surface. They are essential for adsorption processes. Macropores, on the other hand, have a small share in the total internal surface area. In sorbents, they become transport routes and allow access to smaller pores [3].

The porous structure influences the properties of the materials. These include sorption capacity, mechanical, thermal and electrical properties. How these properties depend on the ratio of open to closed pores or vice versa. Also important are the shape of pores, the distribution of their dimensions and spatial arrangement in the material.

The most important parameters characterizing the structure of porous materials are: density, volume porosity, permeability, specific surface.

The action of corrosive agents may lead to an increase in moisture in the material [4]. This applies especially to chemical and biological agents. The latter cause biological corrosion of materials caused by fungi and bacteria. Biological corrosion is a multi-stage and complex process of destroying materials. Biodeterioration is a general reduction in the quality of building materials due to biological agents. Biodeterioration has been noticed not only in sewage systems, submarine pipelines, bridge pillars, oil and gas pipelines and offshore platforms [5, 6]. But also in buildings and flats where people live. In the first group of objects, the reason is usually the action of sulfur bacteria *Thiobacillus sp*., which produces biogenic sulfuric acid. In contrast, the second group of objects is primarily exposed to mold (filamentous) fungi. Of course, in both cases the cost of repair and maintenance is very high. Appropriate conditions, such as optimal temperature, increased relative humidity, can cause microorganisms to grow on building materials. In addition to them, the development of microorganisms can be affected by: high concentration of carbon dioxide, chloride ions and other salts, sulfates and small amounts of acids.

As a result of mould fungi metabolic products are released. These include volatile products, organic acids, water and other chemical compounds. Volatile compounds such as aldehydes, ketones and alcohols create a musty-mould aroma with high permeability to the environment [7]. The material is usually moist as a result of water secretion by fungi into the building material environment. On their surface, microorganisms form a biofilm. Biofilm is formed by complex, multicellular structures in which numerous microbial cells are surrounded by a layer of mucus [8]. It also accumulates water, which allows fungi to survive adverse external conditions.

Each microorganism has its own requirements for a minimum substrate moisture content that will initiate growth and further growth. The growth of microorganisms and the destruction of building materials begins with a water activity (aw) of about 0.7 in the material [9]. *Penicillium chrysogenum* belongs to the group of moderate xerophils for which the minimum moisture content is 0.78 to 0.81 and even 0.85. *Cladosporium herbarum* belongs to the group of weak xerophils for which the minimum moisture content is 0.85 to 0.88. The fungi are classified into three groups of first, second and tertiary colonizers with the minimum water activity. This division is conventional. *Penicillium chrysogenum* is contained as a primary colonizer with water activity, i.e. low water humidity below 0.8 and relative air humidity below 80% and *Cladosporium herbarum* belongs to secondary colonizers at 0.80 to 0.9 and relative air humidity within the range of 89–90% [10].

Biodeterioration is a specific type of biological corrosion that causes deterioration in the performance of building materials as a result of the interaction of two processes of biodegradation of building materials and mycotoxic contamination of the environment [11]. As a result of the microbial metabolism, water is released, which penetrates and influences the building materials. The moisture content of building materials depends on the ambient temperature, the type of material and its structure, e.g. the pore structure and the content of hygroscopic additives. Different building materials can absorb different amounts of water [12]. For a cement mortar, mass humidity is in the range of 1.8÷5.6 [%], and water content expressed in kg/m3 is from 36 to 112, for relative air humidity f = 70÷90%. Due to the fact that the mortar in the state of hygroscopic equilibrium is characterized by mass humidity of 1.8–5.6 [%].

Usually, the absorbability of building materials is lower than the porosity. It is connected with the fact that water is not able to get inside closed pores and in the case of pores with large diameters it does not fill them, only moisturizes the walls. In the case of microorganisms, mass humidity is very often close to absorbability. The durability of materials will be affected by the pore size distribution, which in turn will affect the diffusion rate of ions. Humidity [11, 13], the possibility of deposition of corrosion products or unsealing structures as a result of the action of microorganisms clearly affect the distribution of pores. In Portland cement concrete, CEM I portland is washed out by water, creating "channels" for easy access to water and other aggressive solutions [14].

Moisture in the form of water vapour or liquid originates from atmospheric conditions, but above all from the metabolic activity of mould. This is due to the environmental effect of 1–2.5% mass. All the rest is produced by microorganisms.

The aim of this paper is an attempt to determine how porosity and pore distribution in cement-polymer mortar, i.e. cement mortar modified with 3% polysiloxane admixture, influences the moisture coming from the activity of filamentous fungi.

## 2. Materials and methods

### 2.1. Materials

*The characteristics of the cement used are presented in Table 1*.

**Table 1:** Chemical composition of the cement used (CEM I 42.5).

Content [%mass.]	CaO	SiO_2_	Al_2_O_3_	Fe_2_O_3_	sulphates SO_3_	MgO	K_2_O	Na_2_O	Cl^-^
CEM I 42.5	63.05	18.74	4.90	3.17	2.8	1.32	0.89	0.13	0.01

Quartz sand according to PN-EN 196–1 standard and drinking water according to EN 1008:2000 standard were used for preparation of mortar samples.

The ratio of w/c in the tested mortar was 0.5. The composition of composites covered by the study is presented in **Table 2**.

**Table 2:** Composition of the tested composite.

Designation	Content [g]			
	Cement	Sand	Water	Polymer Modifier
CMPSi/3%	100	300	48.5	3

The test elements were 20x20x160 mm beams made of CEM I standard mortar modified with 3% polysiloxane latex. The prepared samples were stored under foil for 24 hours. After removal from the mould, the samples matured under the foil for 2 days and then at the temperature of +20°C ± 2°C and relative humidity of 60% ± 5% (PN-EN 12190:2000: Products and systems for the protection and repair of concrete structures—Test methods—Determination of compression strength of repair mortar) [15]. The samples were then conditioned under laboratory conditions for 28 days.

### 2.2. Corrosive environment

The most common mould fungi in objects infected with biological corrosion include *Cladosporium*, *Aspergillus*, *Penicillium*, *Alternaria*, *Fusarium*, *Mucor*, *Trichoderma* [16,17]. Therefore, two types were selected for the study, i.e. *Cladosporium* as dominant and *Penicillium* as common.

Growth medium in the form of modified malt extract Agar M-8927 (MEA) from Biochemika company was used for the growth of mould fungi. The medium consisted of MEA, water and glycerine in amounts of 31.28, 1000, 2.35 grams, respectively. The medium described above was used for isolation, detection and determination of filamentous fungi. The final pH of the medium was 4.6 at 25°C [17].

Pure fungal cultures were imported from the collection of pure microbial cultures (LOCK) from the Institute of Fermentation and Microbiology Technology in Łódź. *Penicillium chrysogenum* (LOCK 0531, streina F00680) and *Cladosporium herbarum* (LOCK 0490, streina E123) were selected. The fungal cultures were transferred to distilled water. The MEA medium was solidified on Petri dishes. Fungi in the form of suspensions were inoculated on the medium by culture method. The whole system was incubated at 25°C and 95% relative humidity for 5 days. Then, with a clean and sterile swab, swab was taken to 10 ml of distilled water (a suspension with the number of spores 1 × 10^6^ spores per ml). This suspension was used to contaminate previously prepared samples made of the tested cement-polymer mortar. The contaminated test elements were placed in a biological climate chamber at 25ºC±1º C and 95%±1% relative humidity [17].

### 2.3. Time od experiments

The tests were performed during 3, 6, 9 and 12 months from the moment of contamination with filamentous fungi. For comparison, reference samples were used, i.e. materials uncontaminated with filamentous fungi.

### 2.4. Tested parameters

#### 2.4.1. Mass moisture (μ_M_)

Determined in accordance with PN-85/B-04500 standard [18], by determination of mass. The tested materials were weighed and then dried at 105°C to a constant weight. The value of mass moisture was determined from the formula:

$$u_{m}=\left(m_{w}-m_{s}\right)\cdot 100\%/m_{s}\;\left[\%mass.\right]$$ (1)

where: m_w_—wet sample mass [g], m_s_—dry sample mass [g]

#### 2.4.2. Water absorption/absorbability

The water absorption was carried out in accordance with ASTM (ASTM C 642) [19]. The samples were dried in a drying oven at 105°C to constant weight. They were then placed in water at 25°C. The test lasted until the difference in mass between the two measurements at 24-hour intervals was not less than 0.5%. The amount of water absorption is calculated from the formula:

$$n_{m}=\left(m_{n}-m_{s}\right)\cdot 100\%/m_{s}\;\left[\%mass.\right]$$ (2)

where: m_n_—sample mass of water saturated material [g], m_s_—sample mass of dry material [g]

#### 2.4.3. Capillary rise of water

The beams are placed vertically in a vessel with water so that they are submerged to a depth of 0.5 cm. As a measure of capillary rise of water the weight increase in grams was assumed. The level of moisture in the samples was determined in grams 1, 3, 6 and 24 hours after immersion in water.

#### 2.4.4. Degree of moisture permeation

Degree of moisture permeation is expressed as a percentage, as a ratio of mass moisture of the material to moisture in the state of full saturation. The degree of moisture permeation indicates the percentage of available water pore volume filled with water at the time of the test [20].

#### 2.4.4. Degree of saturation with water

Degree of saturation with water is expressed as a percentage and means the ratio of volume absorbability (water absorption) to porosity.

#### 2.4.5. Porosity

The porosity was determined using the mercury porosimeter Quantachrome Poremaster Nova1000e. This method allows to determine the porosity and pore size distribution in the radius range from 4 to 30000 nm. The test involves filling the pores of the material with mercury at increasing pressure. The procedure for this test is described in detail in [21,22]. Two small samples with a diameter of 10 mm and a length of 10–15 mm are taken from the tested materials. Each sample weighed about 2g. The samples were then dried in the oven at 105–110°C for 24 hours. The next step was to store the samples in a desiccator until testing. The test results are presented in the form of graphs showing the relationship between the volume of the pores and the diameter. For this purpose, the Poromaster program is used. The mercury porosimetry test was also determined: Pc—total porosity [%], V_tot_.—total pore volume [cm^3^/g], W—volume shares of dominant pore populations, determined from the curves of pore size distribution population.

## 3. Results

The results of moisture parameters are presented in two separate tables. For *Penicillium chrysogenum* (abbreviation P.ch.) in **Table 3** and for *Cladosporium herbarum* (abbreviation C.h.) in **Table 4**. The results contained in the tables are the average value from six measurements. The distribution of the results shall not exceed 0.1%. Partial results from moisture and absorbability were presented in [23,24].

**Table 3:** Moisture results for the fungus species *Penicillium chrysogenum*.

Parameter	Time [month]					
	0	3	6	9	12	
mass moisture [%mass.]	2.48	8.12	8.02	8.27	7.89	[17,18]
capillary rise of water [24h %mass.]	1.50	1.78	2.08	2.29	2.40	
absorbability [%mass.]	6.27	8.21	8.03	8.33	8.46	[17,18]
degree of moisture permeation [%]	39.56	98.85	99.81	99.27	93.17	
degree of saturation with water	0.280	0.437	0.425	0.387	0.428	

**Table 4:** Moisture results for the fungus species *Cladosporium herbarum*.

Parameter	Time [month]					
	0	3	6	9	12	
mass moisture [%mass.]	2.48	7.72	7.62	7.85	7.97	[23,24]
capillary rise of water [24h %mass.]		1.77	1.76	1.78	1.81	
absorbability [%mass.]	6.27	7.61	7.62	7.99	8.18	[23,24]
degree of moisture permeation [%]	39.56	101.46	100.04	98.24	97.41	
degree of saturation with water	0.280	0.447	0.373	0.445	0.597	

**Fig 1:**
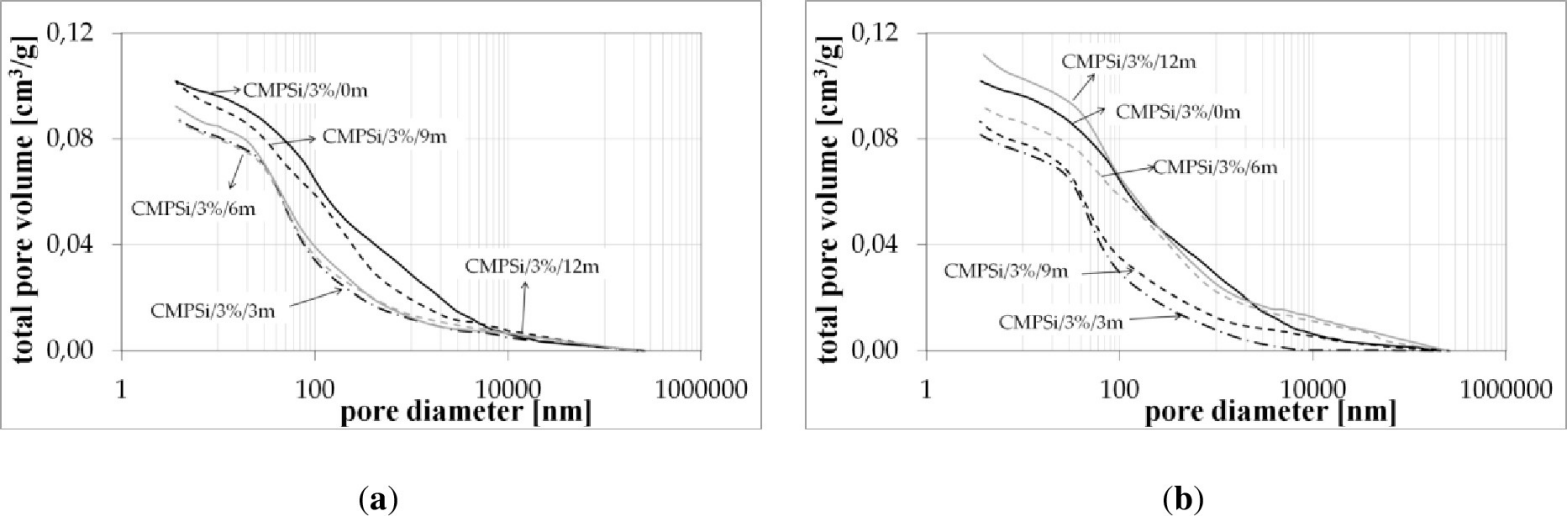
Cumulative curves of pore volume distribution in CMPSi mortar as a function of their diameters in the studied range of pore size for fungal contamination: (a) *Penicillium chrysogenum*; (b) *Cladosporium herbarum* [23].

**Fig 1** presents the results of porosimetry in the form of a cumulative curve graph pore volume distribution in CMPSi mortar.

The curves obtained in the mercury porosimeter (MIP) study allowed to determine the ranges of pores, which were collected in **Table 5**. These pores to a large extent took part in corrosion caused by the action of filamentous fungi. Taking into account the discrepancy in the classification of pores and their nomenclature [4], a simplified division can be assumed. The analysis of the graphs shows that the most important pores were three ranges: below 10, 10–100, 100-5000nm. Therefore, a simplified division of pore size was applied. Then the structure will contain gel pores <10nm, pores associated with crystal products 10-100nm, capillary pores 100-1000nm and macropores >1μm [25].

**Table 5:** Volume shares of dominant pore populations.

Mortar/ quantity/ contamination /time	W [cm^3^/g]						V_tot_	Porosity
	10	10–100	100–5000	<5000	10–5000	Total	[cm^3^/g]	[%]
CMPSi/3%/reference	0.006	0.034	0.052	0.010	0.092	0.102	0.102	22.38
CMPSi/3%/P.ch/3m	0.007	0.048	0.025	0.007	0.081	0.088	0.089	18.79
CMPSi/3%/C.h/3m	0.007	0.046	0.029	0.000	0.081	0.081	0.081	17.01
CMPSi/3%/P.ch./6m	0.008	0.048	0.023	0.009	0.079	0.087	0.087	18.88
CMPSi/3%/C.h./6m	0.007	0.032	0.040	0.014	0.079	0.093	0.093	20.40
CMPSi/3%/P.ch./9m	0.011	0.033	0.047	0.010	0.091	0.101	0.101	21.50
CMPSi/3%/C.h./9m	0.009	0.048	0.023	0.007	0.079	0.086	0.087	17.97
CMPSi/3%/P.ch./12m	0.008	0.046	0.031	0.007	0.085	0.092	0.092	19.79
CMPSi/3%/C.h./12m	0.009	0.047	0.040	0.015	0.096	0.112	0.112	13.70

**Fig 2:**
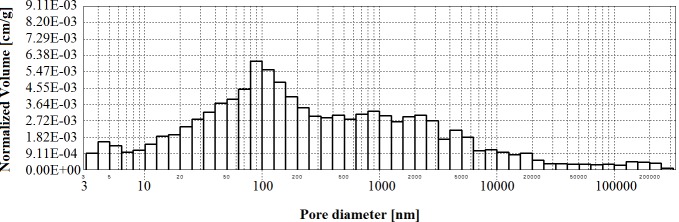
Histogram of pore volume in CMPSi mortar as a function of their diameters in the studied pore size range.

**Fig 3:**
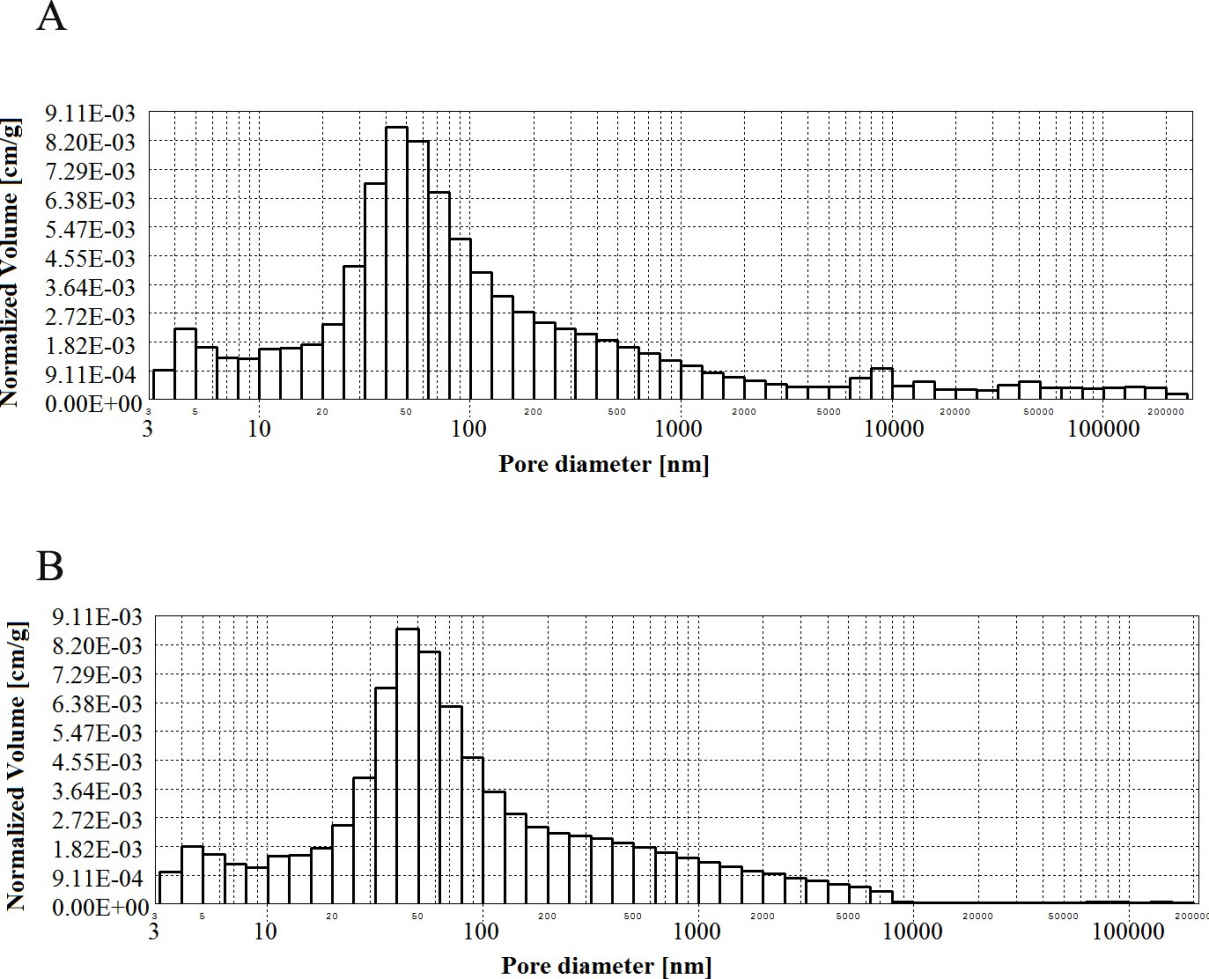
Histogram of pore volume in CMPSi mortar as a function of their diameters in the studied pore size range for contamination (a) *Penicillium chrysogenum*; (b) *Cladosporium herbarum*, after 3 months of exposure.

**Fig 4:**
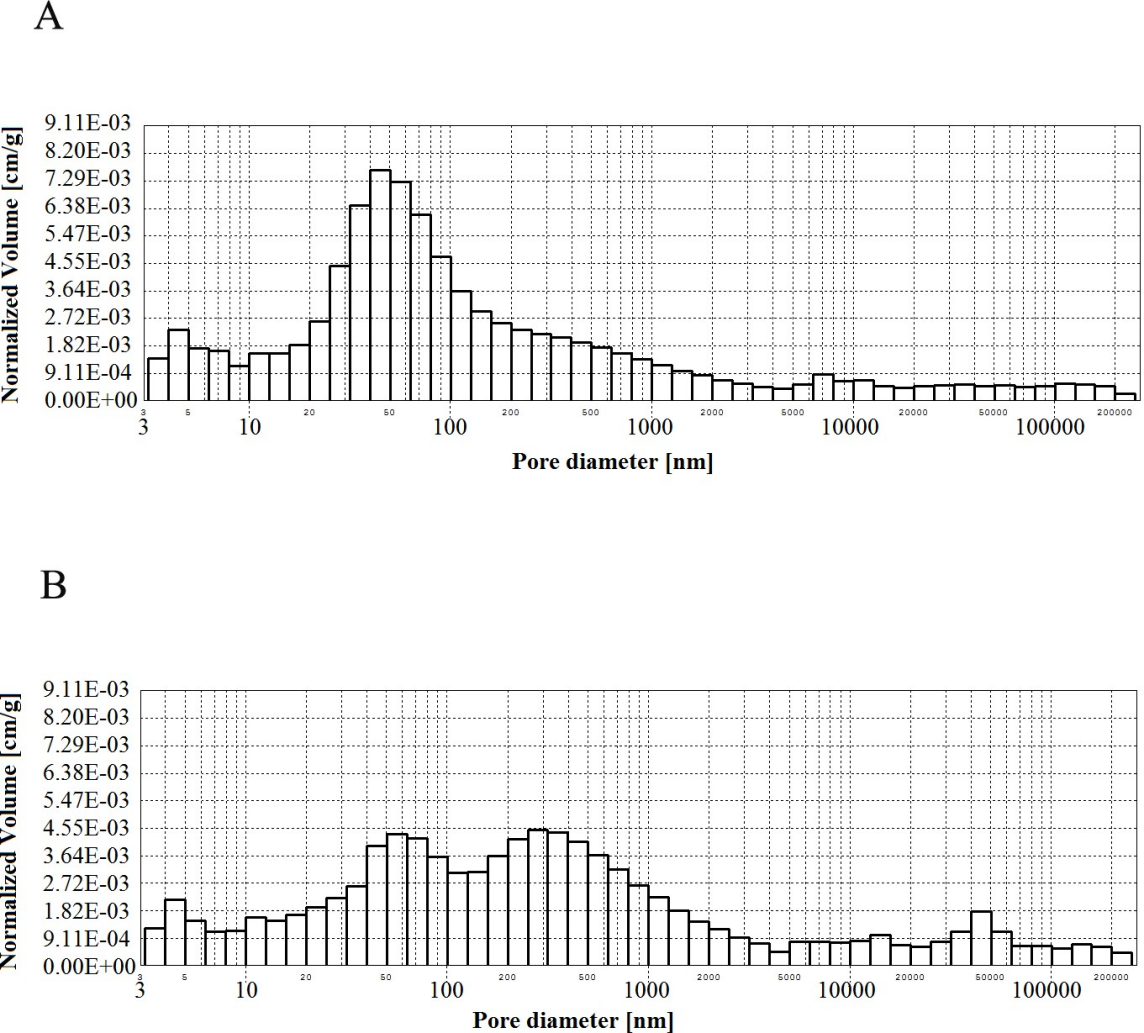
Histogram of pore volume in CMPSi mortar as a function of their diameters in the studied pore size range for contamination (a) *Penicillium chrysogenum*; (b) *Cladosporium herbarum*, after 6 months of exposure.

**Fig 5:**
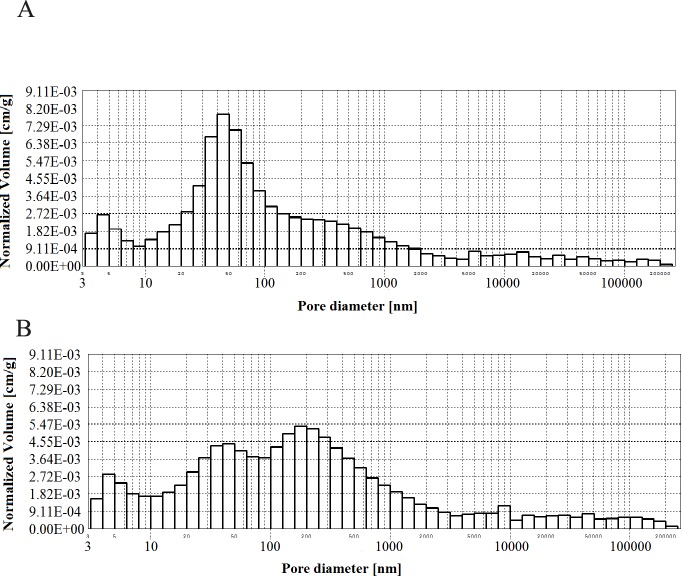
Histogram of pore volume in CMPSi mortar as a function of their diameters in the studied pore size range for contamination (a) *Penicillium chrysogenum*; (b) *Cladosporium herbarum* after 9 months of exposure.

**Fig 6:**
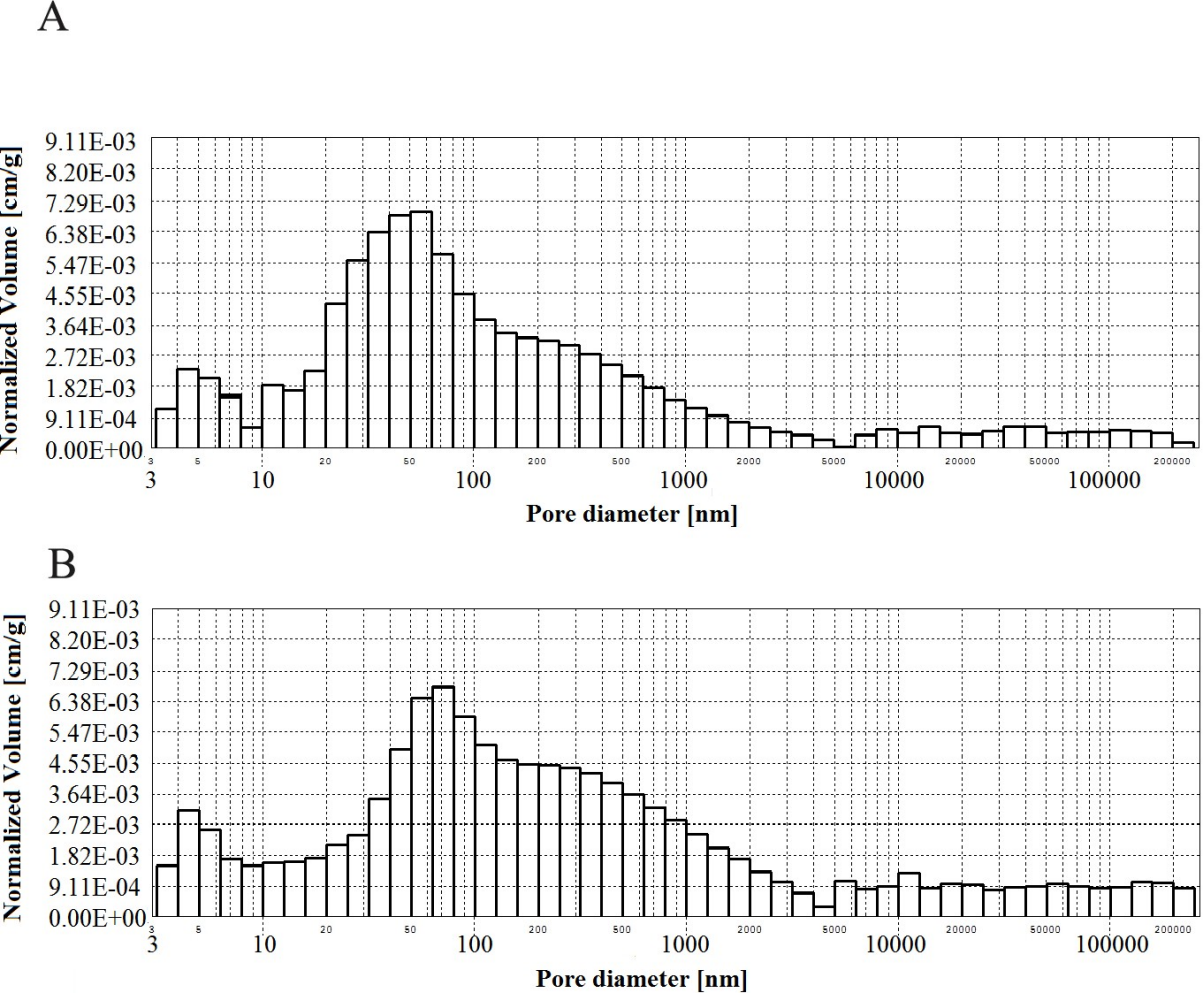
Histogram of pore volume in CMPSi mortar as a function of their diameters in the studied pore size range for contamination (a) *Penicillium chrysogenum*; (b) *Cladosporium herbarum*, after 12 months of exposure.

**Figs 2–6** present histograms showing pore volumes in CMPSi mortar.

## 4. Discussion

The action of mould fungi increases the moisture content of the material during the whole period of exposure to these environments. However, for both *Penicillium* and *Cladosporium* there is a slight decrease within 6 months from the beginning of the experiment. Mortars contaminated with *Penicillium* for 9 months show the highest mass moisture. After 12 months, the materials contaminated with *Cladosporium* show a higher mass moisture content. In relation to the standard mortar, the mass moisture increases by more than 5% mass.

Mass absorbability does not exceed mass moisture. In one case, i.e. when contaminated with a fungus of the *Cladosporium* species, it is at the same level after 6 months and exceeds for the same fungus after 3 months. However, the admixture content causes significant changes. For a fungus of *Cladosporium* species it increases during the whole experimental period. However, in the case of mortars contaminated with *Penicillium* a temporary decrease occurs at 6 months of exposure. A similar situation occurred in the case of mass moisture. Comparing the absorbability of contaminated mortars to the standard one can see that it increases for both fungi by about 2 and over 2% mass. suitable for *Cladosporium* and *Penicillium* fungi.

Moistening of the mortar can be a gradual process, as well as a process changing over time. Moisture levels and fungi content do not necessarily correlate well with building materials [26].

The analysis of capillary rise of water shows that during the whole experimental period the capillary rise of water increases and the highest value is obtained after 12 months. This is the case for both fungi species. However, much higher values are obtained for the genus *Penicillium*.

Analysis of the internal structure of the material allows to determine the type of pores, their quantity and general porosity. Cement-polymer mortar modified with polysiloxane latex did not completely seal the structure with 3% of the admixture. The admixture did not stop the development of microorganisms.

Practically all corrosive environments caused that the most important pores were two compartments: 10–100, 100-5000nm. In general, this was the case for all the exposure times to both fungi species. However, in the case of standard mortar the number of pores of 10-100nm was smaller and the number of pores of 100-5000nm was greater. In the case of contaminated mortars, in most cases the ratio was the opposite. However, there were two cases, i.e. C.h./6m and P.ch./9m, where it was similar to the standard mortar. The porosity of the structure for the 3% admixture ranged from 17 to 24%. The primary porosity (of the reference material) is sealed by the corrosive action of mould fungi. Sealing occurred primarily due to the presence of microorganisms and corrosion products. However, with time it starts to increase. The structure starts to unseal again. For a graphical representation of the internal structure, differential and cumulative curves of the pore distribution are shown in Fig 1. The total volume of pores behaves similarly to porosity. The pore volume and volume shares of the dominant pore populations are shown in **Table 5** and **Figs 2–6** (histograms). The histogram graph allows you to replace the differential curves of the pore volume distribution. The volume of pores decreased from 0.102 for standard mortar and decreased over the next 6 months to increase to the same value after 9 months. However, after 12 months it decreases again. This occurs in the structure of the mortar contaminated with *Penicillium chrysogenum*. It seems that after 9 months the corrosion products are dissolved or some of the fungi die off. The structure of mortars contaminated with *Cladosporium herbarum* behaves differently. The pore volume increases for 6 months, but slightly decreases after another 3 months. Then, in 12 months from the start of the exposure, the value of the standard mortar increased. Histogram charts are included in the paper to better illustrate the behavior of particular pore sizes of mortars contaminated with mould.

Analyzing the effect of moisture on the structure, it can be observed that when *Penicillium chrysogenum* fungus contaminates the pores with a decrease or increase in their volume, the same moisture and absorption behaviour is observed. However, in the case of a fungus of *Cladosporium herbarum* species, when the moisture content of the pores increased, the moisture content decreased and vice versa. It wasn't until 12 months that both values increased.

**Tables 1** and **2** also include two parameters, i.e. the degree of water permeation and the degree of water saturation. The first of these parameters is characteristic for the moisture content of ceramic walls. An attempt was made to adapt this parameter to cement mortars. However, the second parameter is the degree of water saturation. This parameter depends on the volume absorbability and porosity of the sample. It means that it depends on the amount of water per volume of material and takes into account possible changes in the structure of pores. It behaves inversely to the porosity of both fungi species.

A change in the degree of moisture permeation behaves similarly to a change in water absorption during the period of exposure to microorganisms. This is probably due to the fact that the mass moisture and mass absorbability levels are similar. The degree of moisture permeation indicates the percentage of pores filled with water. In other words, it indicates the percentage of available pore volume filled with water at the time of mortar testing. In case of contamination of *Cladosporium herbarum* during 3 and 6 months, the value exceeds 100%. This is probably due to the presence of biofilm on the surface. This means that the biofilm has not been completely removed before the moisture and absorbability test.

## 5. Conclusion

Biodeterioration is a specific type of biological corrosion that causes deterioration in the performance of building materials as a result of the interaction of two processes of biodegradation of building materials and mycotoxic contamination of the environment. As a result of the microbial metabolism, water is released, which penetrates and influences the building materials. The moisture content of building materials depends on the ambient temperature, the type of material and its structure, e.g. the pore structure and the content of hygroscopic additives. Different building materials can absorb different amounts of water.

Moisture in the form of water vapour or liquid originates from atmospheric conditions, but above all from the metabolic activity of mould. This is due to the environmental effect of 1–2.5% mass. All the rest is produced by microorganisms.

The structure of cement mortar modified with 3% polysiloxane latex shows an influence on moisture parameters. The structure changes during the whole time of the action of shredder fungi. An attempt was also made to adjust two new moisture parameters, which are also combined with porosity and pore volume. They allow to evaluate the influence of porous structure and its influence on various properties of building materials. Both degrees, moisture permeation and water saturation still need to be fine-tuned, but they seem promising parameters for determining the influence of moisture from microorganisms on the structure of materials contaminated with them.

The presence of microorganisms increases the amount of water in the materials that they settle. This significantly affects the structure of the material. In the case of cement-polymer mortar, where 3% polysiloxane admixture was used, the material was not completely sealed. It is worth to conduct research on the influence of more admixture on the contamination of cement and polymer mortars with moulds. It is also worthwhile to study the aspect of the influence of the above mentioned influence on the growth of microorganisms.

## Supporting information

S1 File (DOCX)
