# Supplementary material for: Influence of pore structure on humidity parameters of cement-polymer mortars contaminated with filamentous fungi
Source: PLoS One. 2020 Apr 9;15(4):e0231347. doi: 10.1371/journal.pone.0231347 (PMC7145099; doi:10.1371/journal.pone.0231347)
Supplement: S1 File — (DOCX) [file pone.0231347.s001.docx]

Elżbieta Stanaszek-Tomal ^1^*

Chair of Building Materials Engineering**,** Faculty of Civil Engineering, PK Cracow

University of Technology, 24 Warszawska Street, 31-155 Cracow, Poland,

[estanaszek-tomal@pk.edu.pl](mailto:estanaszek-tomal@pk.edu.pl)

ORCID: 0000-0003-2677-2167
